# Absorption, metabolism, and excretion of [^14^C]ponatinib after a single oral dose in humans

**DOI:** 10.1007/s00280-017-3240-x

**Published:** 2017-02-09

**Authors:** Yihua E. Ye, Caroline N. Woodward, Narayana I. Narasimhan

**Affiliations:** ARIAD Pharmaceuticals, Inc., 26 Lansdowne St, Cambridge, MA 02139 USA

**Keywords:** Pharmacokinetics, Radiolabeled, Ponatinib, Human metabolism

## Abstract

**Purpose:**

Ponatinib is a novel tyrosine kinase inhibitor (TKI) specifically designed to inhibit native and mutated BCR–ABL. In the United States, ponatinib has received accelerated approval for adults with T315I-positive chronic myeloid leukemia (CML) or T315I (gatekeeper mutation)-positive, Philadelphia chromosome-positive, acute lymphoblastic leukemia (Ph + ALL), and patients with CML or Ph + ALL for whom no other TKI therapy is indicated. The objective of this phase 1, mass balance study was to evaluate the absorption, metabolism, and excretion of [^14^C]ponatinib in healthy subjects.

**Methods:**

A single 45-mg [^14^C]ponatinib dose was administered orally to six healthy male volunteers, and absorption, metabolism, and excretion were assessed.

**Results:**

86.6 and 5.4% of the dose was recovered in feces and urine, respectively, during days 0–14 postdose. Median time to maximal plasma radioactivity was 5 h and mean terminal elimination half-life of radioactivity was 66.4 h. Ponatinib and its inactive carboxylic acid metabolite M14, the two major circulating radioactive components, accounted for 25.5 and 14.9% of the radioactivity in 0–24 h pooled plasma, with elimination half-lives of 27.4 and 33.7 h, respectively. Major metabolites in urine were M14 and its glucuronides, which, together with other M14-derived metabolites, represented 4.4% of the dose; ponatinib was not detected in urine. In feces, major radioactive components were ponatinib, M31 (hydroxylation), M42 (*N*-demethylation), and four methylated products accounting for 20.5, 17.7, 8.3, and 8.4% of the radioactive dose, respectively.

**Conclusions:**

Ponatinib was readily absorbed in humans, metabolized through multiple pathways and was eliminated mostly in feces.

**Electronic supplementary material:**

The online version of this article (doi:10.1007/s00280-017-3240-x) contains supplementary material, which is available to authorized users.

## Introduction

The use of tyrosine kinase inhibitors (TKIs) that target BCR–ABL has markedly improved the outcomes in patients with chronic myeloid leukemia (CML) [1]. Second-generation TKIs, including nilotinib, dasatinib, and bosutinib, are frequently effective in patients who have developed resistance to first-line imatinib [1, 2]. The best-characterized mechanism of resistance to first- and second-generation TKIs is often mediated by mutations in the ABL kinase domain that disrupt TKI binding. A number of such resistance mutations have been characterized, among which the gatekeeper mutation T315I is uniformly refractory to first- and second-generation TKIs [3–5]. Ponatinib (AP24534) is a novel TKI that was developed using a computational and structure-based approach [6–8] to optimally inhibit native BCR–ABL, as well as mutated forms of the protein, including the T315I gatekeeper mutant [3–5, 7]. Preclinical in vitro studies have demonstrated that ponatinib is a pan-BCR–ABL inhibitor, and can suppress the emergence of any single mutation at a concentration (40 nM) that can be achieved in patients [7, 9].

In phase 1 [9] and phase 2 (PACE) [10] trials, daily oral doses of 45 mg ponatinib demonstrated a positive benefit–risk in CML and Philadelphia chromosome-positive acute lymphoblastic leukemia (Ph + ALL) patients who experienced failure of prior TKIs, and these data supported the initial approval of ponatinib in the United States and the European Union. Ponatinib is the only approved oral TKI with clinical activity against the T315I mutant [7]. Single-dose and steady-state pharmacokinetic profiles of ponatinib have been characterized in the phase 1 study [9]. Ponatinib was readily absorbed following a 45-mg oral dose, with the steady-state maximal plasma concentration (*C*_max_) of 145 nM observed at 4–8 h (median time to *C*_max_ was 4.8 h), and a mean terminal elimination half-life (*t*_1/2_) of approximately 22 h [9].

The objective of this phase 1, open-label, mass balance study was to evaluate the absorption, metabolism, and excretion of [^14^C]ponatinib after administration of a single oral 45-mg dose in healthy male subjects. Herein, we provide detailed quantitative information about the total dose excreted, routes of excretion, and distribution and structural characterization of metabolites in plasma, urine, and feces.

## Materials and methods

### Materials

The following nonradiolabeled reference compounds were provided by the Chemical and Process Development Department, ARIAD Pharmaceuticals, Inc. (Cambridge, MA, USA): ponatinib (AP24534), AP24600 (M14), AP24592 (M19), AP32318 (M23), AP25047 (M34), AP24734 (M36), and AP24567 (M42) (for structures, see Online Resource 5). The purity of the reference compounds was >95% as examined by liquid chromatography/mass spectrometry (LC/MS). Solvents and reagents, all of analytical grade, were purchased from commercial manufacturers.

**Fig. 1:**
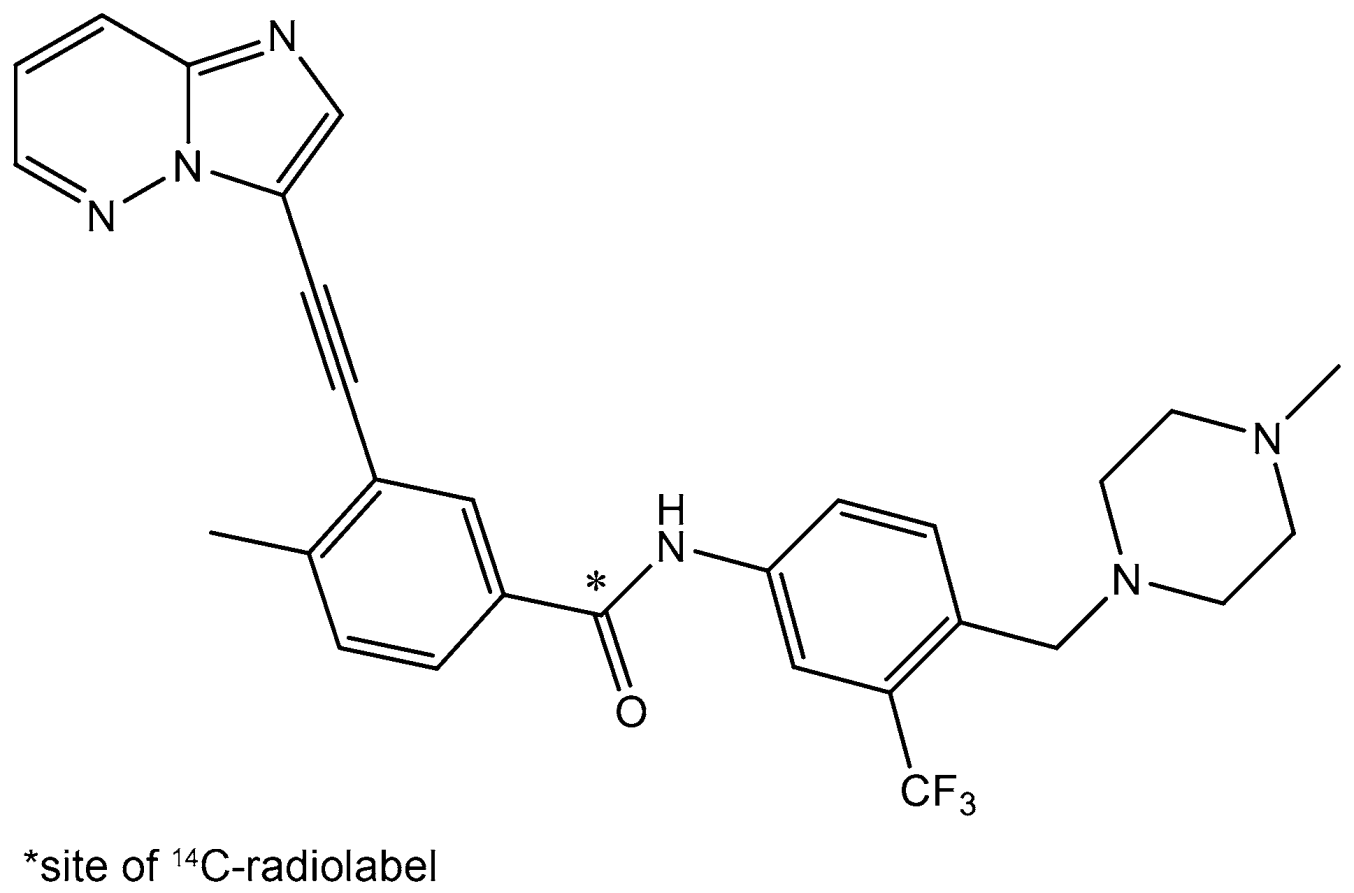
Structure of [^14^C]ponatinib

[^14^C]Ponatinib hydrochloride (Fig. 1) was synthesized (Aptuit LLC, Kansas City, MO, USA) and packed into size-1 gelatin capsules (ABC Laboratories, Inc., Columbia, MO, USA) under Good Manufacturing Practices; each capsule contained ponatinib hydrochloride equivalent to 15 mg of free base (36.7 µCi of radioactivity, specific activity 2.447 µCi/mg). The radiochemical purity of [^14^C]ponatinib hydrochloride was 99.8%.

### Subjects and drug administration

Recruiting of healthy subjects, housing, administration of [^14^C]ponatinib, and collection of blood and excreta were completed at Celerion (Lincoln, NE, USA). The protocol and informed consent for this study were reviewed and approved by the Celerion Institutional Review Board. Informed consent was obtained from all individual participants and the study was conducted in accordance with the ethical standards of the Declaration of Helsinki.

Six healthy male volunteers, 25–45 years of age, with a mean body weight of 83.3 kg (range 64.5–94.4) and a mean body mass index of 26.9 kg/m^2^ (range 20.2–29.9) participated in the study. Each subject received a single oral dose of 45 mg [^14^C]ponatinib (three 15-mg capsules, 110.1 μCi total) with 240 mL of water, in the fasted state on the morning of day 1. Whole blood, urine, and feces samples, and toilet tissue wipes, were collected up to the time of discharge (11–14 days postdose, varied by subject). The subject release criterion was defined as follows: the combined urine and fecal radioactivity excretion for two consecutive days should fall below 1% of the dose administered. Most patients were discharged 11–14 days following dose administration.

### Sample collection, storage, and shipment

Detailed procedures on sample collection, storage, and shipment are described in Online Resource 1. A brief summary is provided here: 12 mL of whole blood was collected from each subject at various time points into K_2_EDTA tubes; 2 mL of whole blood was aliquoted for total radioactivity (TRA) analysis, and the rest of the blood was centrifuged to obtain plasma for TRA analysis, metabolite identification, and liquid chromatography with tandem mass spectrometry (LC/MS/MS) quantitation of ponatinib and two metabolites (M14 and M42). Urine and feces collected from each subject were pooled at predose and every 24 h thereafter. Fecal homogenates were prepared by adding water (four times the feces weight) and homogenizing. Following collection, samples were stored at −80 °C and shipped from the clinical site over dry ice to the following laboratories: plasma, to Advion Biosciences, Inc. (Ithaca, NY, USA) for quantitation of ponatinib and *N*-desmethyl metabolite M42 (AP24567); whole blood, plasma, urine, and feces, to ABC Laboratories for determination of TRA; and plasma, urine, and feces, to ARIAD Pharmaceuticals, Inc. (Cambridge, MA, USA) for metabolite profiling and quantitation of M14.

### Determination of TRA

TRA analysis was carried out at ABC Laboratories. The radioactivity in plasma (duplicate per time point) and urine samples (triplicate per time point) was quantitated by direct counting in a Beckman LS6000SC or LS6500 liquid scintillation counter (PerkinElmer Life and Analytical Sciences, Boston, MA, USA). Whole blood (duplicate per time point) and fecal homogenate (triplicate per time point) samples were oxidized, and the resultant ^14^CO_2_ was trapped in Carbosorb (Calgon Carbon Corporation, Pittsburgh, PA, USA) in combination with Permafluor, followed by liquid scintillation counting to determine the radioactivity. Individual samples were counted for 5 min for whole blood and fecal matrices, and 10 min for plasma and urine matrices. Predose blood, plasma, urine, and feces samples served as respective control samples to determine and subtract the background radioactivity in the analysis.

### LC/MS/MS quantitation of ponatinib and metabolites M14 (AP24600) and M42 (AP24567) in plasma

Ponatinib and M42 (AP24567) were determined using a validated LC/MS/MS method previously described [11]. M14 was quantitated using a different LC/MS/MS method (Online Resource 2). The pharmacokinetic analysis was carried out using WinNonlin^®^ v6.4 (Pharsight Corp., Mountain View, CA, USA).

### Sample preparation of plasma, urine, and feces for metabolite profiling

Structural characterization of the metabolites and quantitative determination (using radioactivity) of ponatinib and metabolites in pooled plasma, urine, and feces were carried out using LC/MS/MS methods coupled with offline radioactivity detection.

For each subject, a 0–24 h pooled plasma sample was prepared according to the scheme outlined in the literature [12]. In addition, several pooled plasma samples across all subjects were prepared at these time points: 5, 10, 12, 36, and 48 h, and every 24 h thereafter until 240 h.

All pooled 0–24, 5, 10, 12, and 24 h plasma samples were processed as follows. Since the radioactivity in the plasma samples was low, unlabeled ponatinib and M14 were added to enhance the recovery. Plasma samples (2.4 mL) were spiked with 1.2 μL of a methanol solution containing ponatinib and M14 (each at 1 mg/mL) followed by 5 mL of ice-cold 1% formic acid in acetonitrile. The samples were vortexed for 30 min and centrifuged at 4500*g* at 4 °C for 6 min. The supernatants were transferred into new polypropylene tubes and kept on ice. The resulting plasma protein pellets were extracted twice to ensure good recovery and the extracts were combined. The combined extracts were loaded onto Ostro sample preparation plates (Waters Corporation, Milford, MA, USA), dried, eluted with 0.2 mL of cold acetonitrile, and all eluates were collected and evaporated under nitrogen (TurboVap LV; Caliper Life Sciences, Hopkinton, MA, USA). The dry residues were reconstituted in 0.1 mL of methanol, and a 0.005-mL aliquot was removed for determination of radioactivity. The remainder of the supernatants were transferred to high-performance liquid chromatography (HPLC) vials and stored at −80 °C until analysis.

Pooled post-24-h plasma samples were extracted using a slightly modified procedure. These samples were spiked with ice-cold 0.1% methanol solution containing deuterated analogs (final concentration, 500 ng/mL) of ponatinib, M42, and M14. Appropriate volumes of formic acid were added to achieve a final concentration of 1%. Then, the samples were warmed to 37 °C and loaded to preconditioned Oasis HLB cartridges (Waters Corporation, Milford, MA, USA). These columns were then washed with water, dried under vacuum, and eluted with methanol. The eluates were collected and evaporated under nitrogen. The dry residues were reconstituted in 0.1 mL of methanol, and a 0.005-mL aliquot was removed for determination of radioactivity. The remainder of the supernatants were transferred to HPLC vials and stored at −80 °C until analysis.

Predose and 0–72-h urine samples (grand pool of all six subjects) were prepared. Urine samples (5 mL) were centrifuged, the supernatant was transferred to a 15-mL polypropylene tube and placed on ice; the residues were extracted with 1 mL of methanol and the methanol extracts were added to the supernatants. The combined extracts were evaporated under nitrogen and reconstituted in 0.2 mL of methanol. A 0.010-mL aliquot of the reconstituted solution was removed for determination of radioactivity, and the remainder was transferred to HPLC vials and stored at −80 °C.

The predose and 0–144-h pooled fecal homogenate samples (grand pool of all subjects) were prepared. One-gram samples of the fecal homogenates were transferred to 50-mL polypropylene tubes, extracted with 4 mL of methanol, and centrifuged (4500*g*). The resulting residues were extracted three more times (once with 2 mL of methanol and twice with 1 mL of methanol containing 1% formic acid) and centrifuged. All supernatants were combined and evaporated under nitrogen, and the residue was reconstituted with 0.5 mL of methanol, vortexed, sonicated, and centrifuged. A 0.010-mL aliquot of the supernatant was removed for determination of radioactivity, and the remainder of the supernatant was transferred to an HPLC vial and stored at −80 °C.

### HPLC-radiochromatographic profiling of ponatinib and its metabolites and structural characterization

A Finnigan Surveyor HPLC system (Thermo Fisher Scientific Inc., San Jose, CA, USA), consisting of an LC pump plus an autosampler and a UV–Vis plus detector, was used mostly for profiling (except the post-24-h plasma samples, which were analyzed using a Dionex HPLC system). A Phenomenex^®^ Gemini^®^ C18, 5-μm, 3.0 × 100-mm column (Phenomenex Inc., Torrance, CA, USA) was used along with a C-12 guard column (4 × 2 mm). LC conditions were as follows: solvent A was 10 mM ammonium bicarbonate in water (pH 10.0) and solvent B was 100% methanol and the flow rate was 0.8 mL/min. The following gradient was employed (B%): 0–5 min (20%), 32 min (42%), 33 min (60%), 55 min (70%), 56–60 min (95%), and 62–70 min (20%).

Reconstituted solvent extracts (0.050–0.10 mL) of plasma, urine, or feces were injected onto the HPLC column. The post-column eluate was split after UV detection into two streams (0.64 and 0.16 mL/min). The 0.64-mL stream was diverted to a fraction collector (Gilson FX 204; Gilson Medical Electronics, Middleton, WI, USA) to be collected in 96-well plates at 0.16 min/well (ScintiPlates; PerkinElmer Life Sciences, Shelton, CT, USA), and the 0.16-mL stream was directed to the mass spectrometer. The solvent in the 96-well plates was removed at 40 °C using a Genevac evaporator (Genevac, Inc., Stone Ridge, NY, USA).

The radioactivity counts per minute (CPM) in each well was determined using a MicroBeta scintillation counter (PerkinElmer Life and Analytical Sciences). The resulting CPM vs. time data were processed using Excel (Microsoft Corporation, Seattle, WA, USA). The CPM in each well was background-subtracted (using the average CPM values of the blank region of the HPLC run). Radiochromatographic profiles were obtained by plotting the resulting net CPM values against time after injection. Radioactive peaks in the chromatographic profiles were identified, and the radioactivity in each peak was summed and reported as a percentage of TRA in the HPLC run.

The remaining eluate stream of 0.16 mL/min from the HPLC runs was diverted to the inlet of the mass spectrometer. The samples were analyzed using a heated electrospray ionization probe. Full-scan positive ion MS data were collected using Finnigan Exactive (MS^1^) or Q-Exactive (MS^1^ and MS^2^) mass spectrometers (Thermo Fisher Scientific Inc.) Exact conditions are described in the Online Resource 3.

## Results

### Concentration–time profiles of TRA, ponatinib, and metabolites in plasma

**Fig. 2:**
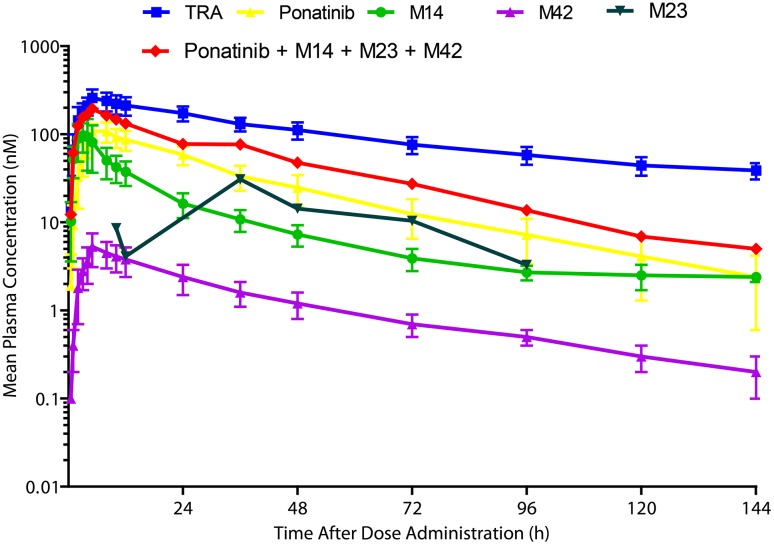
Mean plasma concentration–time profiles of TRA, ponatinib, and metabolites M14 (AP24600), M23 (AP32318), and M42 (AP24567). *Error bars* represent SD; *n* = 6. TRA in plasma was determined using liquid scintillation counting. Ponatinib and its metabolites (M14 and M42) were quantitated using LC/MS/MS methods, whereas M23 was quantitated by radiochromatography [M23 (nM) = ponatinib concentration (nM) × radioactivity ratio of M23/ponatinib]. *LC*/*MS*/*MS* liquid chromatography with tandem mass spectrometry. *SD* standard deviation, *TRA* total radioactivity

The concentration–time profiles of TRA, ponatinib, and metabolites M14, M23, and M42 are illustrated in Fig. 2, and the pharmacokinetic parameters are listed in Table 1. TRA in plasma was determined using liquid scintillation counting. Ponatinib and its metabolites M14 and M42 were quantitated using LC/MS/MS methods, whereas M23 was quantitated by radiochromatography. *C*_max_ of TRA, ponatinib, M14, and M42 in plasma was observed at 2–12 h postdose and maximal levels of M23 were observed substantially late at 36 h. At 144 h postdose, plasma TRA was ~39 nM, whereas ponatinib, M14, M23, and M42 levels were below 2.4, 2.4, BQL (3 nM), and 0.2 nM, respectively. The elimination curves of ponatinib, M14, M23, and M42 are nearly parallel, indicating that their terminal elimination half-lives are very similar. The TRA appeared to decrease in plasma with a longer half-life than that of the parent drug. This was likely due to the contribution of non-identifiable radioactive degradation products slowly released from deep body compartments (ponatinib has a large volume of distribution, *V*_z_/*F* = 1101 L).

**Table 1:** Pharmacokinetic parameters of TRA, ponatinib, and metabolites M14 and M42 in human plasma

Pharmacokinetic parameters^a^	TRA	Ponatinib	M14	M42
*C*_max_, nM (% CV)	251.6 (24.6)	109.6 (23.1)	5.0 (40.9)	107.2 (36.6)
AUC_0–144_, h nM (% CV)	13,841.9 (18.8)	3476.1 (27.9)	169.0 (28.6)	1606.4 (25.0)
AUC_0–∞_, h nM (% CV)	17,526.3 (19.7)	3571.8 (29.1)	180.9 (27.7)	1795.2 (25.5)
*t*_1/2_, h (± SD)	66.4 (13.5)	27.4 (5.5)	33.7 (8.6)	56.0 (39.7)
*t*_max_, h (range)	5 (5–5)	5 (5–12)	5 (5–8)	2 (2–4)

*AUC*_*0–144*_ area under the plasma concentration–time curve from time 0–144 h of last quantifiable concentration, *AUC*_*0*–∞_ area under the plasma concentration vs. time curve from time 0–infinity, *C*_*max*_ maximal observed plasma concentration, *CV* coefficient of variation, *SD* standard deviation, *t*_*1*/*2*_ terminal elimination half-life, *t*_*max*_ time to maximal plasma concentration, *TRA* total radioactivity

^a^All pharmacokinetic parameters are geometric means except *t*_max_ values, which are medians; all pharmacokinetic parameters are based on analysis of 0–144 h plasma

### Excretion and mass balance in urine and feces

**Fig. 3:**
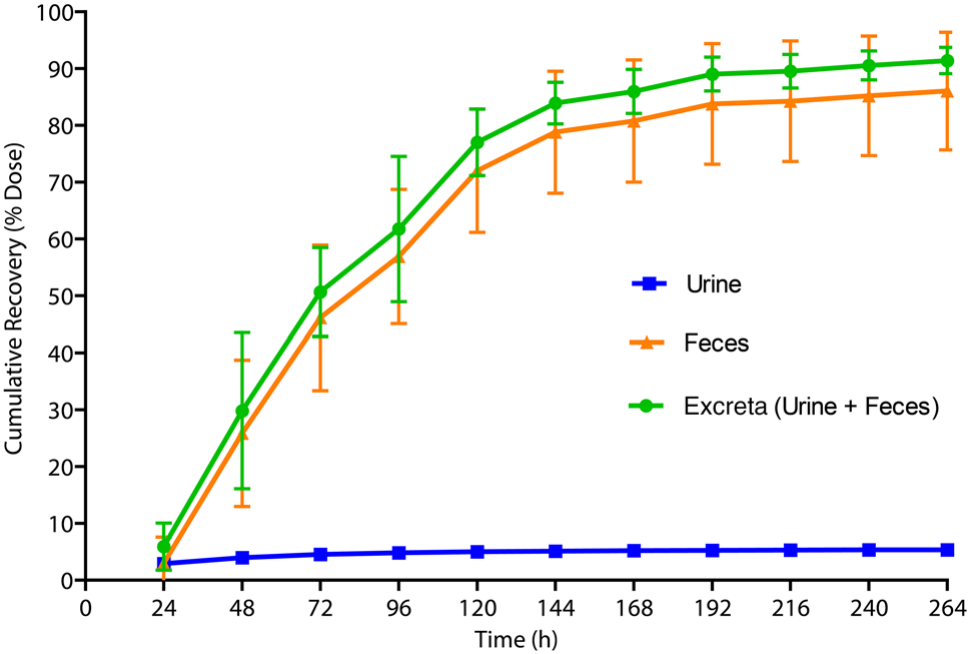
Cumulative recovery of radioactive dose in excreta up to 264 h. *Error bars* represent SD; *n* = 6. TRA in urine was determined using liquid scintillation counting. TRA in feces was determined by oxidization followed by liquid scintillation counting. *SD* standard deviation, *TRA* total radioactivity

The cumulative recovery of radioactive dose in urine, feces, and total dose excreted is presented in Fig. 3. The total radioactive dose recovered during 0–336 h was 92.0%, with 86.6 and 5.4% of the dose recovered in feces and urine, respectively. The cumulative recovery in feces during 0–144 h represented 91% of the TRA in feces. Therefore, metabolite profiling of feces samples was carried out using 0–144 h pool. The urinary metabolite profiling was carried out using the 0–72-h pool as it contained 84% of the total urinary TRA.

### Metabolite characterization by LC/MS/MS

**Fig. 4:**
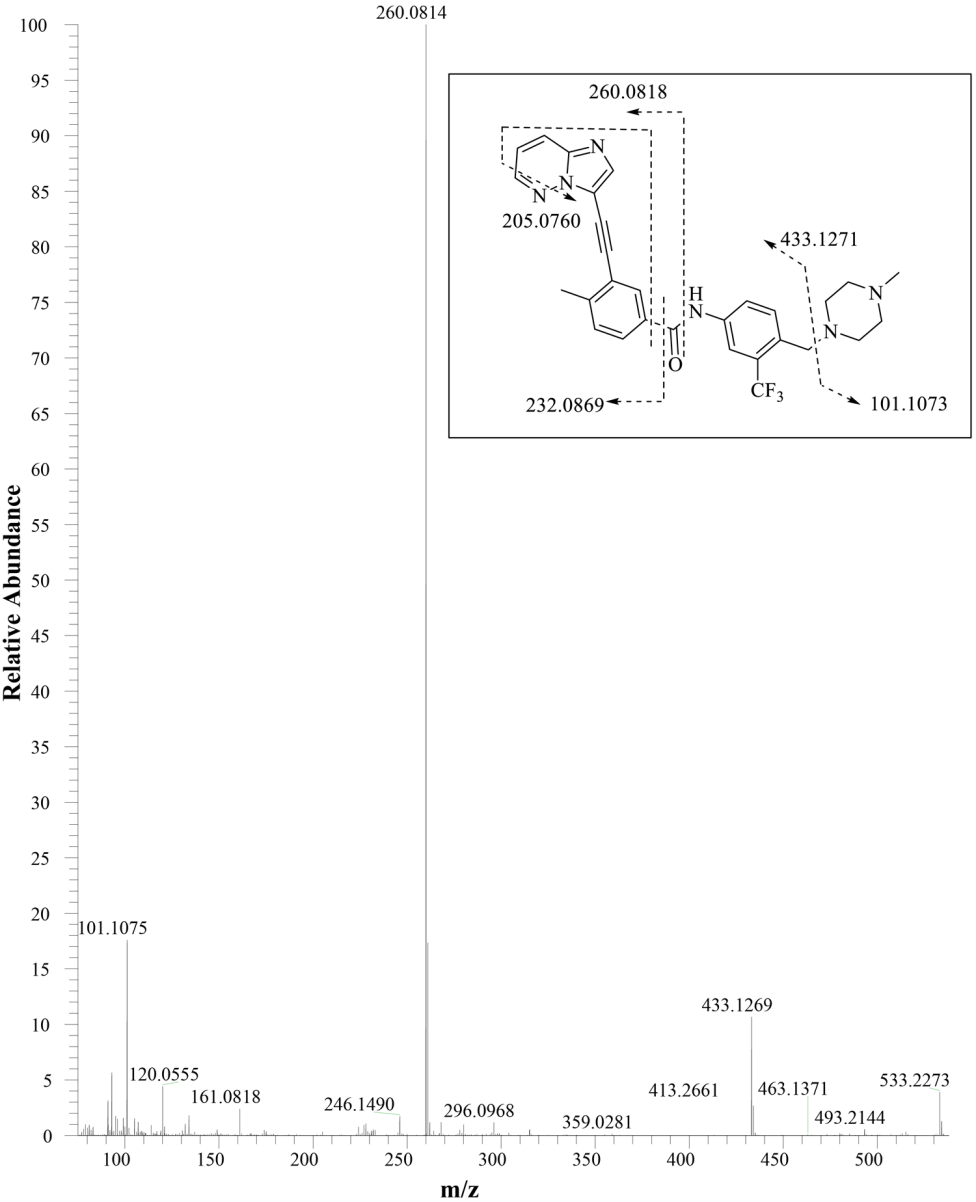
MS^2^ product ion spectrum of ponatinib. *Inset: m*/*z* values displayed in the fragmentation scheme are based on theoretical exact mass of the fragments

**Fig. 5:**
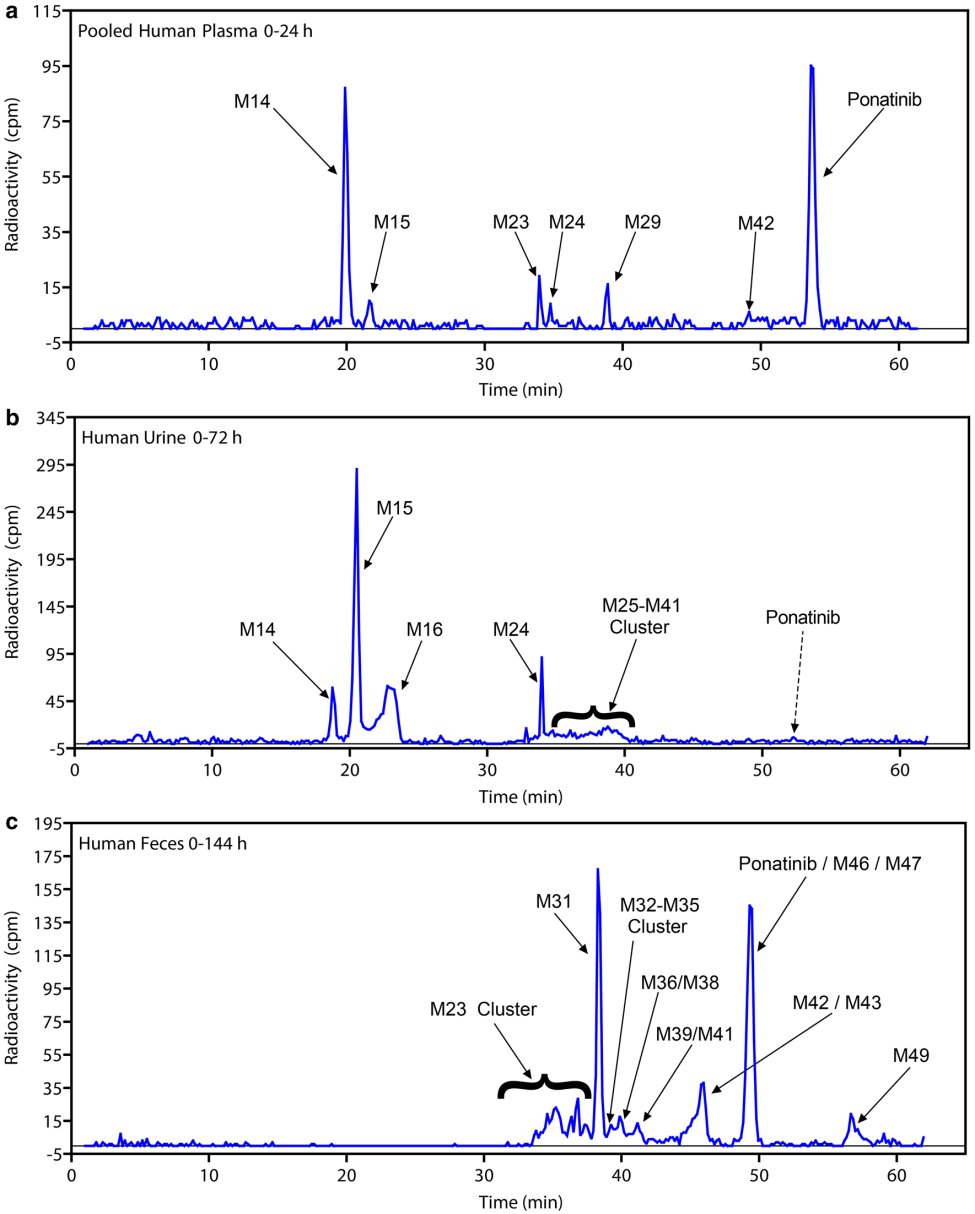
Biotransformation profiles of [^14^C]ponatinib in pooled **a** plasma, **b** urine, and **c** feces. *cpm* counts per minute

**Fig. 6:**
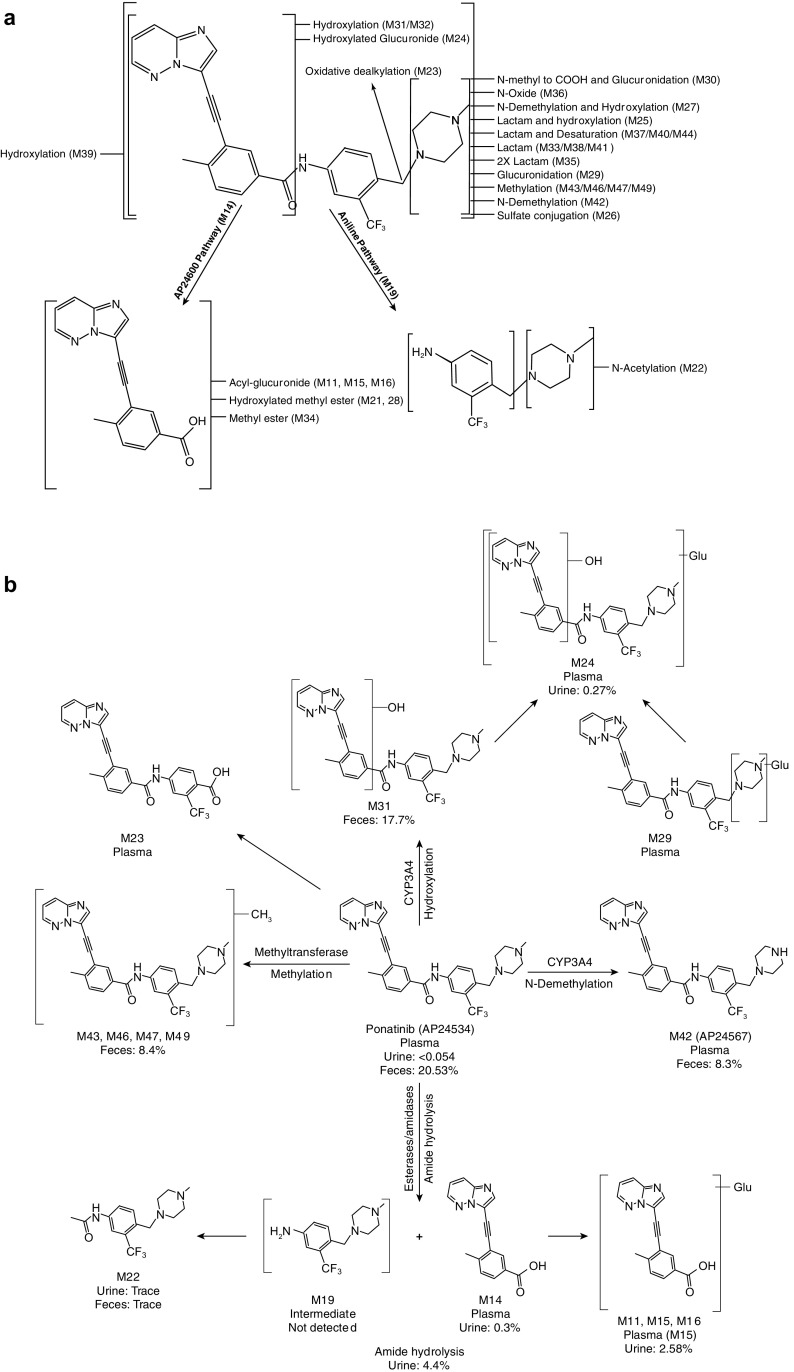
**a** Proposed biotransformation pathways of ponatinib in humans and **b** proposed major biotransformation and elimination pathways of ponatinib in humans

The metabolite structures were characterized by mass spectral fragmentation patterns (Online Resource 5) and, where possible, were confirmed by comparison of chromatographic retention times and mass spectra of the reference standards (AP24600 [M14], AP32318 [M23], AP25047 [M34], AP24734 [M36], and AP24567 [M42]). The MS^2^ fragmentation pattern of ponatinib (AP24534) is shown in Fig. 4. The molecular ion of ponatinib [MH]^+^ was observed at *m*/*z* 533.2278, and the main fragment ions were observed at *m*/*z* 433.1269, 260.0814 (100% abundance), 232.0855, 205.0749, and 101.1075. The fragment ion at *m*/*z* 433.1269 was formed by the neutral loss of *N-*methylpiperazine moiety (100 amu). The amide bond cleavage produced a major fragment ion at *m*/*z* 260.0814, and further fragmentation of *m*/*z* 260.0814 resulted in fragments of *m*/*z* 232.0855 and 205.0749 by successive losses of CO (28 amu) and HCN (27 amu). The ion at *m*/*z* 101.1075 is from the protonated *N*-methylpiperazine group. The fragmentation pattern of ponatinib was compared with those of its metabolites for identification of metabolites (Online Resource 5). The proposed metabolite structures were supported by exact mass measurements of their [MH]^+^ ions. The metabolites of ponatinib predominantly underwent fragmentation analogous to that of the parent drug, which allowed localization of structural changes in the metabolites. Metabolite profiling of plasma, urine, and feces is shown in Fig. 5, and the proposed metabolic pathways are depicted in Fig. 6. The metabolites fall into two broad categories: metabolites of intact ponatinib (oxidation, demethylation, sulfation, glucuronidation, methylation, and extensive metabolism of the piperazine moiety) and those of amide hydrolysis product M14 and its further metabolites (glucuronidation and esterification).

### Metabolite profiling of plasma

A representative radiochromatographic profile of metabolites in pooled 0-24 h plasma after administration of [^14^C]ponatinib to healthy human volunteers is presented in Fig. 5a. Preliminary radiochromatographic profiling and mass spectral data analyses indicated ponatinib and its amide hydrolysis acid metabolite M14 as the two largest radioactive components in the plasma. Radioactivity recovered during preliminary extractions was in the range of 50–70%. Having established the presence of M14 and ponatinib in plasma, a modified extraction scheme was developed to maximize the extraction of radioactivity. Unlabeled ponatinib and M14 were spiked into plasma samples prior to extraction, which resulted in an increase in radioactivity recovery (92.4%). Since unlabeled standards were spiked, this method did not affect the quantitation by radiochromatography. Moreover, this modified procedure had little impact on the interpretation of the data because the qualitative identification of ponatinib and M14 was established before this modified method was adopted.

Metabolite profiles of pooled plasma samples taken at several time points during 0–144 h were qualitatively similar among the six subjects (Table 2 and Online Resource 4). In the 0–24-h pooled plasma, ponatinib accounted for 25.5% of the TRA. The metabolites M14 and M14 glucuronide (M15) accounted for 14.9% and 3.4% of the plasma radioactivity, respectively (Table 2). Ponatinib despiperazinyl acid (M23), ponatinib glucuronide (M29), hydroxyponatinib glucuronide (M24), *N-*desmethyl ponatinib (M42), and ponatinib *N-*oxide (M36) accounted for 7.0, 6.0, 2.1, 0.5, and 0.5% of the plasma radioactivity, respectively. In addition to M14, the amide hydrolysis would yield a putative aromatic amine (M19). However, M19 was not observed in any human samples, but only trace levels of acetylated M19 (M22, nonradioactive) were observed in MS traces in human plasma, urine, and feces. Radiochromatograms of the 5-, 10-, and 12-h plasma extracts are shown in Online Resource 4A, 4B, and 4C, respectively, and the corresponding data are presented in Table 2. In these samples, ponatinib, the glucuronides M14 and M15, M23, and ponatinib glucuronide M29 were also observed (similar to the 0–24 h plasma).

**Table 2:** Percent distribution of ponatinib and its metabolites in human plasma

Metabolite ID	Retention time (min)	% Distribution in plasma^a^							
		5 h	10 h	12 h	0–24 h	36 h	48 h	72 h	96 h
M14	16.6–20.1	29.0	10.8	12.8	14.9	2.4	–	–	–
M15	20.2–21.8	2.0	2.4	–	3.4	–	–	–	–
M23	32.1–34.2	–	3.4	2.4	7.0	3.9	5.4	10.7	6.0
M24	33.5–35.0	–	–	–	2.1	–	–	–	–
M29	38.2–39.2	3.0	4.3	6.0	6.0	–	–	–	–
M36	38.2–42.5	~0.5	~0.5–1.0	~0.5–1.0	~0.5–1.0	–	–	–	–
M42	44.8–49.8	~0.5–1.0	~0.5–1.0	~0.5–1.0	~0.5–1.0	–	–	–	–
Ponatinib	47.8–53.2	42.4	36.3	51.5	25.5	4.2	9.4	12.8	13.2

*TRA* total radioactivity

^a^Only radioactive peaks containing ≥2.0% TRA in the metabolite profile of plasma are listed, except M36 and M42, which were major in vitro metabolites. Radiochromatographic profiles of plasma beyond 96-h collection did not reveal any distinct peaks. “–” values less than 0.5% are not reported

As a result of low levels of radioactivity in post-24-h plasma samples, quantitation by radioactivity was limited to ponatinib and two of the metabolites (Online Resources 4D–4I; Table 2). The 36-h plasma radiochromatogram showed three prominent peaks that were identified as M14, M23, and ponatinib. Radiochromatograms of 72- and 96-h postdose plasma showed only M23 and ponatinib. No distinct radioactive peaks were observed in the extracts of plasma samples collected at 120 and 144 h postdose. Using accurate mass data and the available synthetic standard, the structure of M23 was unequivocally established as ponatinib *N*-despiperazinyl acid.

### Metabolite profiling of urine

Representative radiochromatographic profile of metabolites in pooled 0–72 h human urine is shown in Fig. 5b, and the percent distributions of ponatinib and its metabolites in human urine are presented in Table 3. Ponatinib was observed only at trace levels in urine. The overall urinary metabolite profile was dominated by M14 and its glucuronides, M15 and M16. In 0–72 h urine, M14, M15, and M16 accounted for 5.6, 28.1, and 19.8% of the TRA, respectively (accounting for 53.5% of the total urinary radioactivity). Hydroxyponatinib glucuronide (M24) contributed 5.1% to the TRA in urine. A cluster of metabolites eluted at 35–41 min comprising several metabolites: M25, M29, M30, M31, M32, M33, M34, M35, M36, M38, M39, M41, and other co-eluting metabolites. Individually, each metabolite in the cluster accounted for approximately 0.1–3.0% of the urinary radioactivity and, together, these metabolites accounted for 14.0% of the TRA in urine.

**Table 3:** Percent distribution of ponatinib and its major metabolites in urine and feces after administration of a single oral 45-mg dose of [^14^C]ponatinib to humans

Matrix [collection interval]	Metabolite ID	Retention time (min)	% Distribution in matrix	% of total radioactive dose
Urine^a^ [0–72 h]	M14	16.6–20.1	5.6	0.3
	M15	20.2–21.8	28.1	1.51
	M16	22.5–24.5	19.8	1.07
	M24	33.5–35.0	5.1	0.27
	M25–M41 cluster^b^	35.0–41.0	14	0.75
	Ponatinib	47.8–53.2	Trace levels	<0.05
Feces^*a*^ [0–144 h]	M23 cluster^c^	32.1–37.6	17.2	14.9
	M31	38.0–38.9	20.4	17.7
	M32–M35 cluster^d^	38.5–39.5	2.1	1.8
	M36/M38	39.2–40.5	3.2	2.8
	M39/M41	40.1–41.5	3	2.6
	M42/M43	44.8–49.8	9.6/3.4	8.3/2.9
	Ponatinib/M46, M47	47.8–54.0	23.7/3.2	20.5/2.8
	M49	55.4–57.5	3.1	2.7

*TRA* total radioactivity

^a^Only peaks containing >1.5% of the TRA were included

^b^M25–M41 cluster: metabolites include M25, M29, M30, M31, M32, M33, M34, M35, M36, M38, M39, M41, and other co-eluting unknown metabolites individually accounting for approximately 0.1–3% of the urine radioactivity

^c^M23 (*N*-despiperazinyl acid) cluster: Metabolites include M23, M24, M25, M26, M27, and other co-eluting unknown metabolites individually accounting for approximately 0.1–3% of the fecal radioactivity

^d^M32–M35 cluster: Metabolites include M32, M33, M35, and other co-eluting unknown metabolites individually accounting for approximately 0.1–1.0% of the fecal radioactivity

### Metabolite profiling of feces

Representative radiochromatographic profile of metabolites in pooled 0–144 h human feces is shown in Fig. 5c, and the percent distribution of ponatinib and its metabolites is presented in Table 3. In 0–144 h feces, ponatinib accounted for 23.7% of the radioactivity. Hydroxyponatinib (M31) and *N-*desmethyl ponatinib (M42) were the two largest radioactive metabolites in human feces, and accounted for 20.4 and 9.6% of the radioactivity, respectively (Table 3). Several metabolite peaks that were not well resolved chromatographically formed a cluster (M23 cluster, eluting at 33.0–37.6 min) and cumulatively accounted for 17.2% of the fecal radioactivity; however, each metabolite from the cluster only contributed approximately 2–3%. Other metabolites observed in feces were M32, M33, M35, M36, M38, M39, and M41 (each <0.5%), and cumulatively, they contributed 2.1% of the total fecal radioactivity. There were four ponatinib metabolites (M43, M46, M47, and M49) with an exact molecular weight of [MH]^+^ 547.2426 differing by 1.7 mmu from a theoretical mass difference for a putative methylated metabolite. Together, these four methylated metabolites contributed 9.7% to the total fecal radioactivity (Table 3). No M14-derived metabolites were detected in feces.

## Discussion

Following oral administration of a single dose of 45 mg [^14^C]ponatinib to healthy volunteers, 92% of the dose was recovered 0–336 h postdose; a majority (86.6%) of the dose was recovered in feces and only a small percentage (5.4%) was recovered in urine. Low levels of radioactivity were observed in plasma even at 264 h postdose plasma, whereas ponatinib, M14, and M42 levels in plasma were at or below detectable limits 144 h postdose.

In 0–24 h human plasma, ponatinib and M14 were the major radioactive components, with M14 present at 14.9% of the plasma TRA. M23, the product of *N*-dealkylation of the despiperazinyl group, was a minor metabolite (7% of plasma TRA). Dealkylation of the piperazinyl group has been observed in an another TKI, imatinib [13]. M42, a major in vitro metabolite (data not shown), was present only at <1% in circulation. The same metabolites were also observed in the post-24-h plasma samples, and no new ponatinib metabolites were observed after 24 h. Both M14 and M23 were shown to be inactive against BCR–ABL in in vitro studies (kinase as well as cellular activity screens) (data not shown). The metabolites in urine were mostly M14 and its glucuronides. M14 was generated due to hydrolysis of the amide bond of ponatinib. Amide hydrolysis has been observed in human studies of flumatinib, a nilotinib analog being tested for its efficacy in patients with CML [14]. Since M14 and derived metabolites accounted for 81% of the TRA in urine (and none in feces), and the total dose recovered in urine was 5.4%, it was estimated that the amide hydrolysis accounted for 4.4% of the dose; therefore, amide hydrolysis represented a minor pathway of ponatinib metabolism. In human feces, ponatinib accounted for 23.7% of the radioactivity and was extensively metabolized, and most of the metabolites were generated from the biotransformation of intact ponatinib, mainly at the piperazine moiety (Fig. 6a). Extensive biotransformation at the piperazine group has also been observed with imatinib in humans [13]. Among the metabolites generated, hydroxyponatinib and *N-*desmethyl ponatinib were the most abundant; several minor metabolites that resulted from two or more metabolic modifications were also identified. Four methylated ponatinib metabolites (M43, M46, M47, and M49) totaling 9.7% (of fecal TRA) were only observed in feces, and these metabolites were likely generated as a result of metabolism of ponatinib by methyl transferases [15, 16]. Nearly all methylation biotransformations lead to fewer polar metabolites, which is also true in the case of ponatinib.

Overall, the proposed in vivo metabolism of ponatinib in humans (in plasma, urine, and feces) (Fig. 6b and Online Resource 5) indicates multiple biotransformation pathways. In vitro metabolism studies, using human liver microsomes and CYP-specific chemical inhibitors, have shown that ponatinib was metabolized mostly by CYP3A4 and to a lesser extent by CYP2D6 and CYP2C8 (data not shown). In addition, clinical drug–drug interaction studies have shown that co-administration of ketoconazole (a potent CYP3A4 inhibitor) increased ponatinib *C*_max_ and area under the concentration–time curve from zero to infinity (AUC_0–∞_) by 78 and 47%, respectively [11]. The major elimination pathways for ponatinib following a single oral administration are presented in Fig. 6b; they are hydroxylation (18.0%), *N*-demethylation (8.3%), methylation (8.4%), and amide bond hydrolysis (4.4%), among which hydroxylation and *N*-demethylation were mostly CYP3A4-mediated. These data indicate that ponatinib was readily absorbed and eliminated through multiple pathways following administration of a single oral dose in healthy male subjects.

## Electronic supplementary material

Below is the link to the electronic supplementary material.

Supplementary material 1 (DOCX 1081 KB)

## Abbreviations

AP24534 (ponatinib): 3-(imidazo[1,2-b]pyridazin-3-ylethynyl)-4-methyl-*N*-(4-((4-methylpiperazin-1-yl)methyl)-3-(trifluoromethyl)phenyl)benzamide hydrochloride
AP24567 (M42): 3-(imidazo[1,2-b]pyridazin-3-ylethynyl)-4-methyl-*N*-(4-(piperazin-1-ylmethyl)-3-(trifluoromethyl)phenyl)benzamide
AP24592 (M19): 4-((4-methylpiperazin-1-yl)methyl)-3-(trifluoromethyl)aniline
AP24600 (M14): 3-(imidazo[1,2-b]pyridazin-3-ylethynyl)-4-methylbenzoic acid
AP24734 (M36): 4-(4-(3-(imidazo[1,2-b]pyridazin-3-ylethynyl)-4-methylbenzamido)-2-(trifluoromethyl)benzyl)-1-methylpiperazine 1-oxide
AP25047 (M34): Methyl 3-(imidazo[1,2-b]pyridazin-3-ylethynyl)-4-methylbenzoate
AP32318 (M23): 4-(3-(imidazo[1,2-b]pyridazin-3-ylethynyl)-4-methylbenzamido)-2-(trifluoromethyl)benzoic acid
AUC: Area under the concentration–time curve
BQL: Below quantitation limit
*C*_max_: Maximum plasma concentration
CML: Chronic myeloid leukemia
CPM: Counts per minute
CV: Coefficient of variation
h: Hours
HPLC: High-performance liquid chromatography
LC/MS: Liquid chromatography/mass spectrometry
LC/MS/MS: Liquid chromatography with tandem mass spectrometry
Ph + ALL: Philadelphia chromosome-positive acute lymphoblastic leukemia
SD: Standard deviation
*t*_1/2_: Terminal elimination half-life
TKI: Tyrosine kinase inhibitor
*t*_max_: Time to maximal plasma concentration
TRA: Total radioactivity
