# Supplementary material for: Absorption, metabolism, and excretion of [14C]ponatinib after a single oral dose in humans
Source: Cancer Chemother Pharmacol. 2017 Feb 9;79(3):507–18. doi: 10.1007/s00280-017-3240-x (PMC5344956; doi:10.1007/s00280-017-3240-x)
Supplement: Supplementary file 1 — Supplementary material 1 (DOCX 1081 KB) [file 280_2017_3240_MOESM1_ESM.docx]

**Online Resource Information:**

**Online Resource 1 Blood Urine and Fecal Sample Collection**

**Blood Sample Collection**

Venous blood samples were collected by venipuncture or optional indwelling catheter into K_2_EDTA tubes. To determine TRA in blood and plasma, and plasma concentrations of unchanged ponatinib and metabolites, approximately 12 mL of blood were drawn at each of the specified time points up to 216 h postdose, and then at 24 h intervals until the release criterion (see methods: Subjects and Drug Administration) was met and the subjects were discharged. After blood collection, the tubes were immediately placed over ice, and the whole blood was then aliquoted (2 mL each) and transferred to a −80 ± 15ºC freezer for TRA analysis by ABC Laboratories, Inc. (Columbia, MO). The rest of the blood (approximately 10 ml) was centrifuged to obtain plasma (approximately 4 ml) within the 30 minutes. Approximately 1 ml plasma was transferred to a polypropylene tube and then kept frozen at −80 ± 15ºC until shipment to ABC Laboratories for analysis. Approximately 0.7 ml plasma was transferred to a polypropylene tube and kept frozen at −80 ± 15ºC until shipment to Advion Biosciences, Inc. (Ithaca, NY) for analysis. The remaining plasma was transferred to a polypropylene tube and kept frozen at −80 ± 15ºC until shipment to ARIAD Pharmaceuticals, Inc. (Cambridge, MA).

**Urine Sample Collection**

All urine voided was collected and stored in containers at 5±3ºC until the collection interval was completed. Subjects voided in the morning prior to study drug administration. For the first 24 h postdosing, each collection period was 6 h (e.g., 0-6, 6-12, 12-18, and 18-24 h postdose); thereafter 12 h (e.g., 24-36, 36-48, 48-60, and 60-72 h postdose) and 24h (e.g., 72-96, 96-120, 120-144, and 144-168 h postdose) collection periods were used until subjects were discharged from the clinical research unit.

Multiple collections of urine during each collection interval from each individual subject were pooled. The urine samples from each collection interval were well mixed, accurately weighed, and assessed for its pH. A 100 ml sample from each collection interval from each subject was aliquoted into a separately labeled container to be sent to ABC Laboratories for radioactivity analysis. An approximately 10 ml sample from each collection interval from each subject on day 8 and each day thereafter was used for TRA analysis at Celerion (Lincoln, NE). All urine samples were kept frozen at −80 ± 15ºC.

**Fecal Sample Collection**

Stool collections began approximately 24 h prior to drug administration until the end of the postdose sampling period. The 24 h predose samples were used as control samples for the radioactivity analysis and metabolite profiling. Feces were collected daily (24 h collection periods) until subjects were discharged from the clinical research unit.

All fecal samples were collected in polypropylene containers; toilet paper was collected separately. The weight of each sample was recorded. Multiple collections of feces during each collection interval from each individual subject were pooled. The feces samples were thoroughly homogenized with water (4X the weight of the feces). A 100 g sample was aliquoted into a separately labeled container to be sent to ABC Laboratories for radioactivity analysis. An approximately 10 g sample from each collection interval from each subject on day 8 and each day thereafter was used for TRA analysis at Celerion. All fecal homogenate samples were kept frozen at −80 ± 15ºC.

**Online Resource 2 LC-MS/MS Quantitation method of M14 (AP24600)**

Aliquots of 30 µl of calibration standards, quality controls, and incurred plasma samples were transferred into a 96-well plate. Then 150 µl of ice-cold acetonitrile containing 50 ng/ml of AP30376 (d6-AP24600, internal standard) were added. The 96-well plate was vortexed for 10 min and then centrifuged at 4750 rpm for 20 min at 4°C. 100 µl aliquots of the supernatants were transferred into another 96-well plate and the samples were analyzed by liquid chromatography tandem mass spectrometry.

**Online Resource 3 Mass Spectrometer Instrument Conditions**

Finnigan Exactive or Q-Exactive mass spectrometers (Thermo Fisher Scientific Inc., San Jose, CA) were used to collect full scan MS^1^ and MS^2^ data.

| **Parameter** | **Exactive MS** | **Q-Exactive MS** |
| --- | --- | --- |
| Ion spray voltage | 4 kV | 3.50 kV |
| Capillary temperature | 350ºC | 320ºC |
| Heater temperature | 350ºC | 320ºC |
| S-Lens | N/A | 60 |
| Sheath gas | 60 | 41 |

**Online Resource 4. Radiochromatographic profile of pooled human plasma following administration of [^14^C]ponatinib to healthy human volunteers**


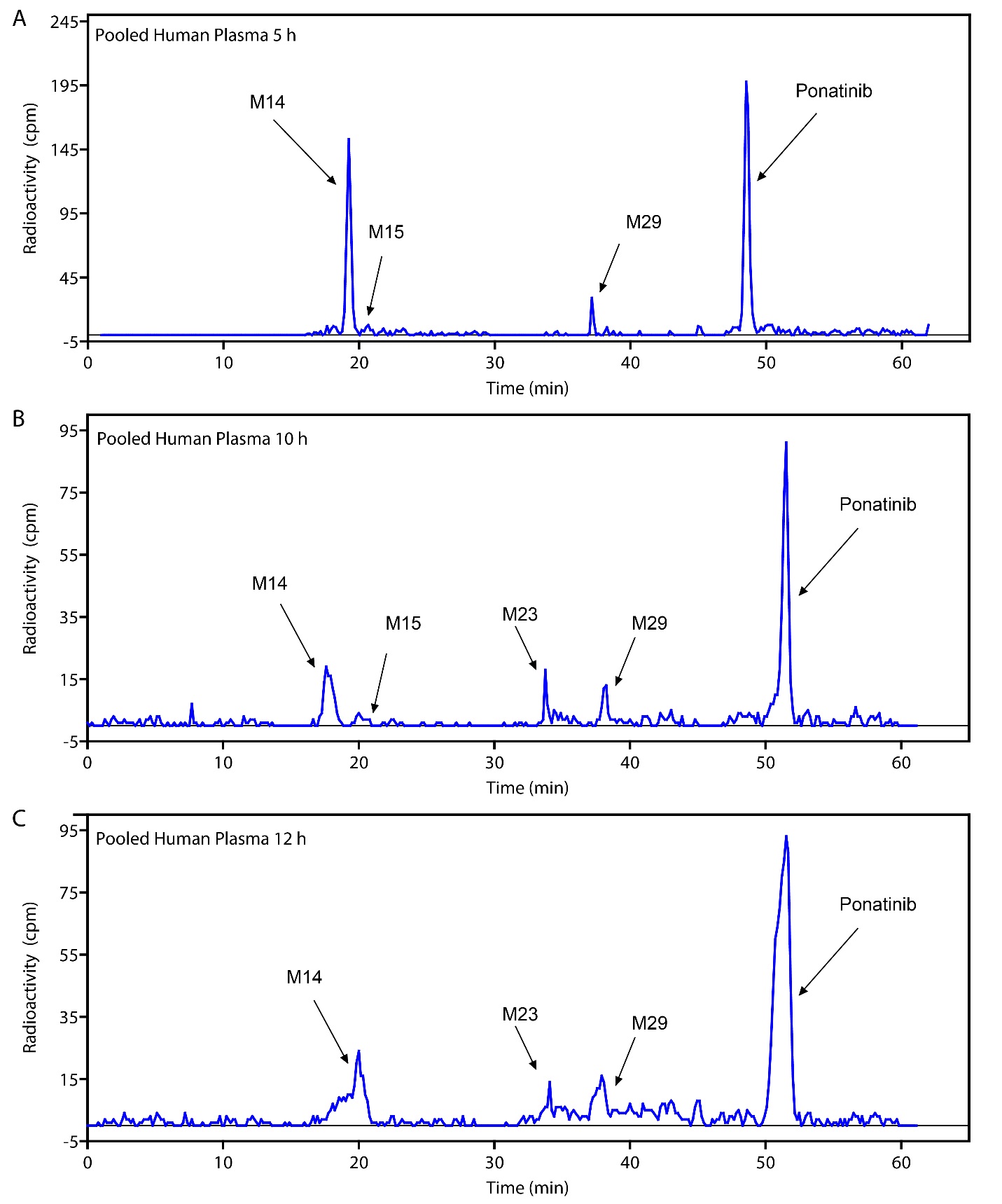


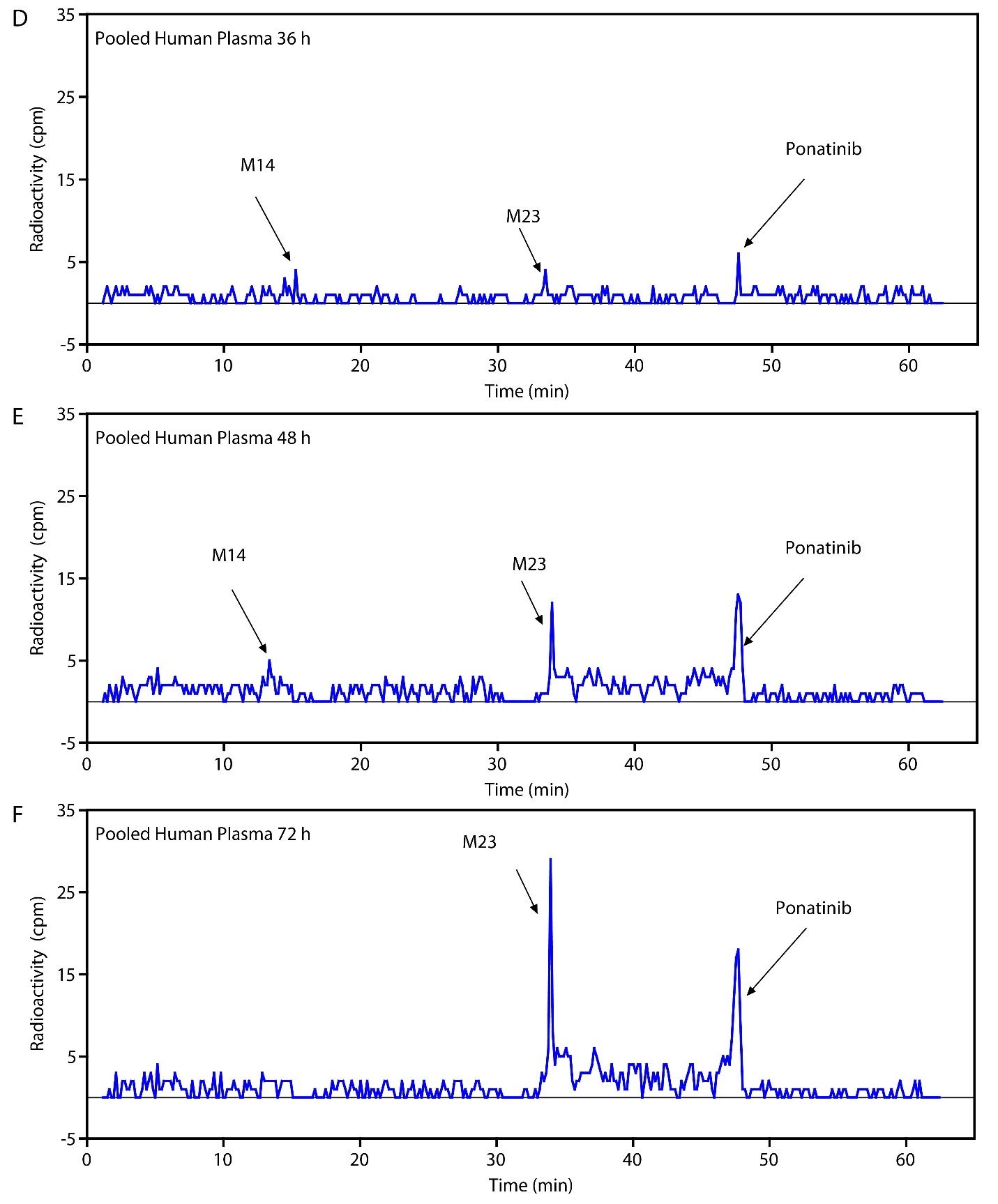


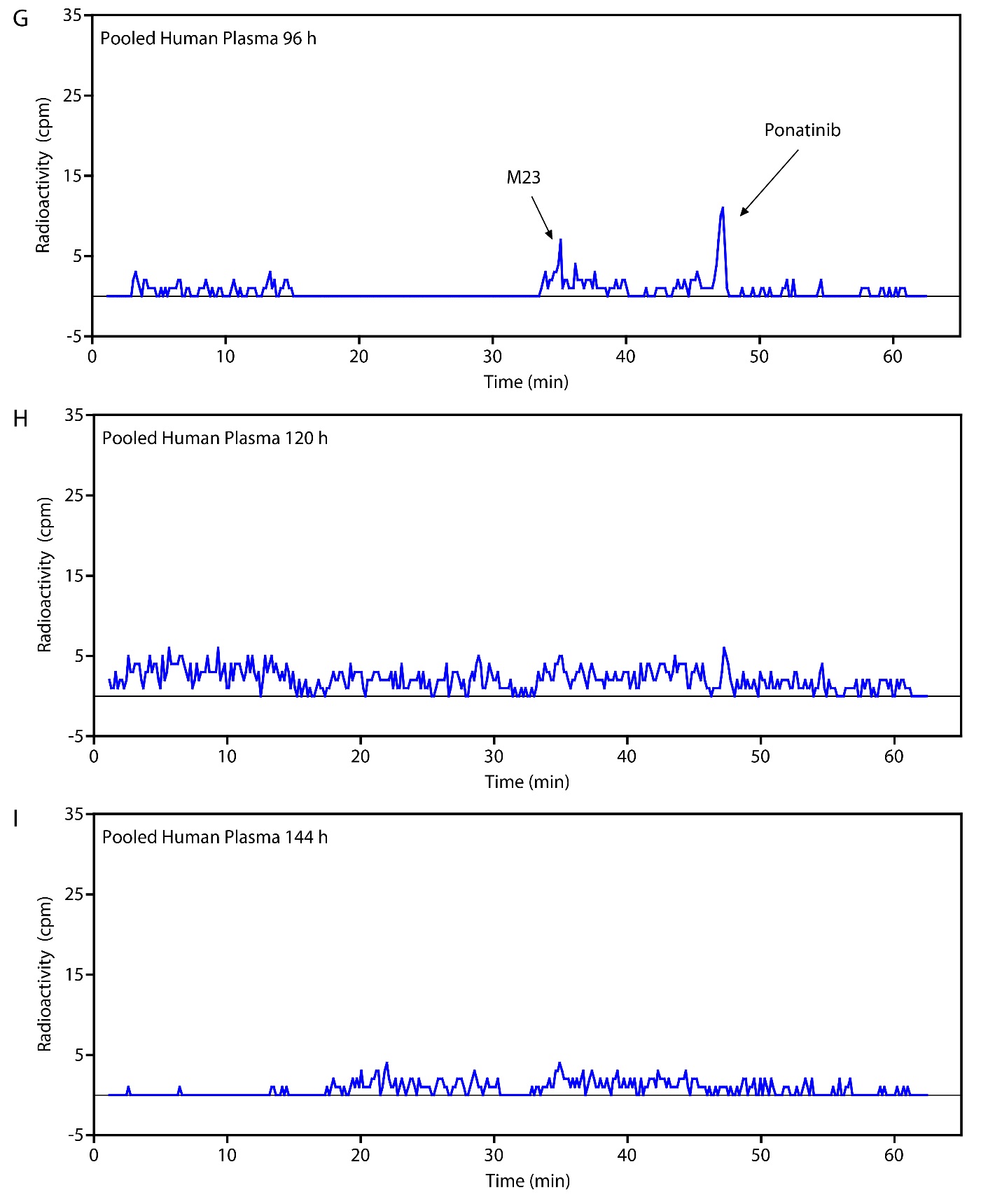


**Online Resource 5. Mass spectral characterization of ponatinib and its metabolites in human plasma, urine, and feces**

| **Metabolite ID** | **Observed [MH]^+^**  **Theoretical Mass Difference (mmu)** | | **Observed Ions,**  ***m/z*** | | **Biotransformation** | | **Proposed Structure and Fragmentation Assignments H^+^** | | **Matrix** | **Structural Characterization** | |
| --- | --- | --- | --- | --- | --- | --- | --- | --- | --- | --- | --- |
| **Ponatinib**  **(AP24534)** | 533.2278 (0.6) | | 433.1255, 260.0803, 232.0855, 205.0749, 101.1070 | | \| Parent \| \| --- \| | |  | | P, U, F | Parent drug: see result (Metabolite Characterization by LC/MS/MS) | |
| **M11, M15, M16** | 454.1252 (0.7) | | 278.0914, 260.0809, 232.0856 | | M14 glucuronides | |  | | P (M15), U | M11, M15, and M16 had a protonated molecular ion [MH]^+^ at *m/z* 454.1252 (176.0325 amu higher than M14) and major product ions at *m/z* 260 and 278 (neutral loss of 176 amu). The exact mass measurement suggested that these 3 metabolites are glucuronide conjugates of M14 (identified as AP24600). | |
| **M14 (AP24600)** | 278.0927 (0.3) | | 232.0854, 205.0747, 127.0534 | | Amide bond hydrolysis of ponatinib | |  | | P, U | M14 had a protonated molecular ion [MH]^+^ at *m/z* 278.0927, which had the same retention time and mass spectral fragmentation pattern as that of the synthetic standard AP24600. | |
| **M19 (AP24592)** | 274.1514 (1.2) | | 174.0525, 101.1073 | | Amide bond hydrolysis of ponatinib | |  | | Reference standard | The amide hydrolysis also would result in an aniline metabolite M19 (AP24592). However, this metabolite was not detected in any matrices. | |
| **M21, M28** | 308.1026 (1.5) | | 294.0859, 276.0755, 248.0806, | | Hydroxy M14-methyl ester | |  | | U, F | M21 and M28 had a protonated molecular ion [MH]^+^ at *m/*z 308.1026 (16 amu higher than that of M34) and major product ions at 294 and 276 (16 amu higher than the major product ions at *m/z* 278 and 260 of M14). The exact mass measurement suggested that M21 and M28 were hydroxylated M14 methyl esters (hydroxylated M34). | |
| **M22*^a^*** | 316.1619 (0.1) | | 216.0631, 174.0525, 101.1074 | | *N-*acetylation of M19 | | ** | | Trace: P, U, F | M22 had a protonated molecular ion [MH]^+^ at *m/z* 316.1619, which was 42 amu higher than M19, the aniline metabolite. This metabolite is consistent with acetylation of M19, the putative amide hydrolysis aniline metabolite. M19 was further metabolized by acetylation and was detected only at trace levels in plasma, urine, and feces. | |
| **M23**  **(AP32318)** | 465.1163 (−0.6) | | 447.1065, 260.0819, 232.0869, 205.0761, 115.0542 | | Oxidative dealkylation of methylpiperazine ring | |  | | P, U, F | M23 had a protonated molecular ion [MH]^+^ at *m/z* 465.1163 and fragment ions at *m/z* 447, 260, 232, 205, and 115. This metabolite is consistent with *N*-dealkylation of the piperazine ring followed by oxidation to a carboxylic acid. | |
| **M24** | 725.2522 (−1.9) | | 549.2205, 449.1213, 276.0764, 248.0815, 101.1074 | | Hydroxy glucuronide of ponatinib | |  | | P, U, F | M24 had a protonated molecular ion at *m/z* 725.2522 and fragment ions at *m/z* 549, 449, 276, 248, and 101. This metabolite is consistent with hydroxylation followed by glucuronidation. | |
| **M25** | 563.2017 (0.4) | | 433.1272, 260.0819, 232.0870, 205.0761 | | Hydroxylation and ketone formation on methylpiperazine moiety | |  | | U, F | M25 had a protonated molecular ion at *m/z* 563.2017 and fragment ions at *m/z* 433, 260, 232, and 205. This metabolite is consistent with bis-oxygenation followed by dehydrogenation. | |
| **M26** | 613.1837 (0.2) | | 533.2287, 260.0807 | | Sulfate conjugation | |  | | U, F | M26 had a protonated molecular ion at *m/z* 613.1837 and fragment ions at *m/z* 533, and 260. The fragment at *m/z* 533 is consistent with loss of sulfonic acid (−80 amu) of the parent ion. This metabolite is consistent with direct sulfation of ponatinib. | |
| **M27** | 535.2062 (0.3) | | 433.1272, 260.0818, 232.0869, 205.0761,103.0866 | | *N-*demethylation and hydroxylation of methyl- piperazine | | ** | | U, F | M27 had a protonated molecular ion at *m/z* 535.2062 and fragment ions at *m/z* 433, 260, 232, 205, and 103. This metabolite is consistent with combination of oxygenation and demethylation. | |
| **M29** | 709.2586 (1.3) | | 533.2281, 433.1273, 232.0868, 260.0819 | | Glucuronic conjugation | |  | | P, U, F | M29 had a protonated molecular ion at *m/z* 709.2586 and fragment ions at *m/z* 533, 433, 232, and 260. The fragment at *m/z* 533 is consistent with loss of glucuronic acid (−176 amu) of the parent ion. This metabolite is consistent with direct glucuronidation of ponatinib. | |
| **M30** | 739.2305 (−2.9) | | 563.2008, 519.2116, 433.1271, 260.0817, 232.0867, 205.0867, 87.0917 | | Methyl to carboxylic acid to glucuronidation | |  | | U, F | M30 had a protonated molecular ion at *m/z* 739.2334 and fragment ions at *m/z* 563, 519, 433, 260, 232, 205, and 87. The fragment at *m/z* 563 is consistent with loss of glucuronic acid (−176 amu) of the parent ion. The fragment ion at *m/z* 563 is consistent with dioxygenation and dehydration. This metabolite is consistent with glucuronidation of a carboxylic acid of ponatinib. | |
| **M31, M32** | 549.2228 (0.8) | | 449.1213, 276.0764, 248.0815, 101.1033 | | Hydroxylation on heterocyclic ring or benzylic hydroxylation | |  | | P, U, F | M31 and M32 had a protonated molecular ion [MH]^+^ at *m/z* 549.2228 (16 amu higher than that of ponatinib) and major product ions at 449, 276, 248, and 101. The fragments at *m/z* 276 and 248 had a molecular weight 16 amu higher than those of ponatinib, consistent with oxygenation of the carboxylic side of ponatinib molecule. | |
| **M33, M38, M41** | 547.2051 (−1.7) | | 433.1255, 260.0803, 232.0855, 205.0749 | | Lactam formation | |  | | U, F | M33, M38, and M41 had a protonated molecular ion [MH]^+^ of *m/z* 547.2051 (14 amu higher than that of ponatinib) and major product ions at 433, 260, 232, and 205, which are similar to those of ponatinib. This metabolite is consistent with oxygenation of the piperazine ring followed by oxidation to a lactam. | |
| **M34**  **(AP25047)** | 292.1078 (−0.3) | | 260.0805, 232.0856, 205.0749 | | Methyl ester of M14 | |  | | U | M34 had a protonated molecular ion [MH]^+^ at *m/z* 292.1078 and major product ions at 260, 232, and 205, which had the same retention times and mass spectral fragmentation patterns as the available reference standard, AP25047, which is consistent with the methyl ester of M14 (AP24600). | |
| **M35** | 561.1838 (−1.8) | | 433.1273, 260.0818, 232.0868, 205.0760, 113.0393 | | Double lactam formation | |  | | U, F | M35 had a protonated molecular ion [MH]^+^ at *m/z* 561.1838 and fragment ions at *m/z* 433, 260, 232, 205, and 113. This metabolite is consistent with bisoxygenation at the piperazine ring and further oxidation to a double lactam. | |
| **M36 (AP24734)** | 549.2228 (0.8) | | 433.1254, 260.0805 | | Ponatinib *N-*oxide on methylpiperazine | |  | | P, U, F | M36 had a protonated molecular ion [MH]^+^ at *m/z* 549.2228 and fragment ions at *m/z* 433 and 260 which had the same retention time and mass spectral fragmentation patterns as the available reference standard M34, i.e., the piperazine *N*-oxide of ponatinib. | |
| **M37, M40, M44** | 545.1891 (−1.6) | | 433.1253, 260.0803, 232.0855, 205.0748, 113.0705 | | Lactam formation and desaturation on methylpiperazine moiety | |  | | U, F | M37, M40, and M44 had a protonated molecular ion [MH]^+^ at *m/z* 545.1891 and fragment ions at *m/z* 433, 260, 232, 205, and 113, which are similar to those of ponatinib. This metabolite is consistent with dehydrogenation of the piperazine ring lactam. | |
|  |  |  |  |  |  |  |  | |  |  |  |
| **M39** | 549.2228 (0.8) | | 433.1254, 260.0805, 232.0870 | | Hydroxylation on piperazine of M42 | |  | | U, F | M39 had a protonated molecular ion [MH]^+^ at *m/z* 549.2228 and fragment ions at *m/z* 433, 260, and 233, which are similar to those of ponatinib. This metabolite is consistent with oxygenation at the piperazine ring of M42. | |
| **M42 (AP24567)** | 519.2097 (−0.3) | | 433.1253, 260.0803, 232.0855, 205.0748 | | Ponatinib-*N-*demethylation | |  | | P, U, F | M42 had a protonated molecular ion [MH]^+^ at *m/z* 519.2097 and fragment ions at *m/z* 433, 260, 232, and 205, which had the same retention times and mass spectral fragmentation patterns as the available reference standard AP24567, i.e., *N*-demethylation at the piperazine ring of ponatinib. | |
| **M43, M46, M47, M49** | 547.2426 (−1.7) | | 433.1268, 260.0815, 232.0867 | | Ponatinib methylation | |  | | U, F | M43, M46, and M47 had a protonated molecular ion [MH]^+^ at *m/z* 547.2426 and fragment ions at *m/z* 433, 260, and 232, similar to those of ponatinib. This metabolite is consistent with methylation of ponatinib. | |
| *^a^*Nonradioactive metabolite, fragmentation determined only by high-resolution mass spectrometry.  F=feces; P=plasma; U= urine. | | | | | | | | | | | |
|  | |  | |  | |  |  |  | | |  |
